# Relationships between cervical sagittal posture, muscle endurance, joint position sense, range of motion and level of smartphone addiction

**DOI:** 10.1186/s12891-023-06168-5

**Published:** 2023-01-23

**Authors:** Maryam Heidary Torkamani, Hamid Reza Mokhtarinia, Mohsen Vahedi, Charles Philip Gabel

**Affiliations:** 1 Department of Ergonomics, University of Social Welfare and Rehabilitation Sciences, Kodakyar Ave.Daneshjo Blvd, Evin, Tehran, 1985713834 PC Iran; 2 Department of Biostatistics and Epidemiology, University of Social Welfare and Rehabilitation Science, Tehran, Iran; 3 Access Physiotherapy, Sunshine Coast, Coolum Beach, QLD Australia

**Keywords:** Smartphone, Addiction, Neck muscles, Endurance, Proprioception

## Abstract

**Background:**

Frequent smartphone use in a pathological way forces the user to adopt a compromised posture. This gradually results in changes to both the postural and musculoskeletal systems. This study’s objectives were evaluation of head posture, muscle endurance, neck range of motion (ROM) and joint position sense in two separate smartphone user groups, one ‘Addicted’, the other ‘Non-Addicted’.

**Methods:**

A sample of convenience (*n* = 60) was recruited from medical students (age 24.57 ± 4.38, 53.3% male) with a history of smartphones use > 2 h/day for 1-year. Based on the cut-off values of the smartphone addiction scale-short version (SAS-SV), participants were entered into each group (cut-off for male ≥ 31, female ≥ 33). Neck muscle endurance time, joint position error and cervical ROM, along with forward head posture parameters of craniovertebral angle (CVA), shoulder angle (SA), sagittal head angle (SHA) and forward head distance (FHD)) were evaluated. A Mann–Whitney test and Spearman correlation coefficient were used to determine the difference between groups and the correlations between variables.

**Results:**

The difference between ‘Addicted’ and ‘Non-Addicted’ groups was confirmed by the values for SAS-SV scores (25.23 ± 5.5 versus 43.9 ± 6.61) (*p* < 0.001). There were statistically significant differences between groups for the CVA and FHD parameters (*p* < 0.001). Further, the neck extensor muscle endurance (97 ± 3.79 versus 74.86 ± 2.23 s), was significantly different between groups (*p* = 0.010) but not after Bonferroni correction. There was no notable difference in the neck flexor muscle endurance, joint position error, SA, and SHA parameters between groups (*p* > 0.05).

**Conclusions:**

There is a positive correlation between smartphone addiction and both decreased extensor muscle endurance and changes in neck postural alignment.

## Background

With the increased prevalence of smartphone usage worldwide, a new transformation has occurred in the area of communications and information exchange among people [1]. The smartphone user rate from 2016 to 2020 increased from 3.6 to 6.5 billion, almost one third of the world’s population [2], and has become one of the major global health challenges of the twenty-first century. Iran is the world’s 12^th^ highest smartphone user with over 52 million in 2021, a 25-fold increase on the two million users noted in 2013 [2].

The smartphone has potential use in a variety of daily activities that includes talking with friends and family, web-surfing, bank transactions, television programs, searching, news and current affairs, webinar participation, listening to music, social communication, and entertainment [3, 4]. Because of its portability, lightness, simplicity, and fast information processing ability, smartphones pose a risk to users for addictive behaviors [5, 6]. Smartphone addiction is considered within the category of ‘behavioral addictions’ in the field of human–machine interaction, sometimes referred to as “technological addictions” [7]. Numerous studies have shown that the consequences of this pathological addiction are negative physical and mental health implications, especially for mental [8] and psychosocial conditions [9–12].

The commonly adopted smartphone user postures are when standing or sitting, at which time the smartphone is held with one hand and the screen touched with the other. Forward head posture (FHP) is a commonly adopted but poor position for smartphone users as most smartphone tasks are performed with neck flexion and the arms out in front of the user to read and touch the screen. This habitual FHP causes an alteration in cervical and thoracic spine curvature with an excessive anterior curve in the lower cervical region and an excessive posterior curve in the upper thoracic region. This postural position occurs to maintain the muscle balance [13] and vision accuracy. Maintaining a static posture and performing a repetitive task are the two most common risk factors that lead to the neck and upper extremity musculoskeletal disorders [14] of reduced muscle flexibility and neck range of motion (ROM) [15, 16].

Smartphones facilitate a static user posture for prolonged periods resulting in decreased muscle excursion. The muscle receptors play an important role in detecting joint movement and position sense for appropriate reflexive and voluntary movements [17]. The cervical muscles high muscle-spindle intensity highlights their significant role in proprioception [18]. Induced fatigue in the neck muscles, from adopting poor postures such as FHP, along with decreased muscle elongation, can affect the gamma motor neurons activation, which eventually alters neck proprioception [18, 19]. In several studies the role of neck endurance, flexibility and proprioception are confirmed as causative for neck pain [20]. Due to the high prevalence of neck pain in smartphone users [21], evaluation of these variables and assessment of their correlation with the level of addiction should provide new information about the probable risk factors for smartphone users. To our knowledge there are no studies which have evaluated the proprioception, neck muscle flexibility and endurance in smartphone users in relation to different levels of dependency. Therefore, this study aimed to investigate the relationship between smartphone addiction and neck joint position sense, muscle endurance, and neck ROM.

## Methods

### Participants

This cross-sectional study was conducted between February and August 2021 at the Department of Ergonomics, University of Social Welfare and Rehabilitation Sciences. A total of 60 volunteer students (age 24.57 ± 4.38, range 18 to 40 years, 53.3% male) were recruited from a sample of convenience within four University Medical centers in Tehran, Iran. Inclusion criteria were a history of smartphone use for more than one year and 2 h per day. Exclusion criteria were a history of neck pain, radicular pain, neurological symptoms, and surgery to the neck and/or upper limbs. All subjects provided written informed consent prior to the study. The study was approved by the ethical committee of the University of Social Welfare and Rehabilitation Sciences, number IR.USWR.REC.1400.035.

### Procedure

Demographic information was collected through a checklist that included sex, age, education, daily duration of smartphone use, areas of discomfort or symptoms when working with a smartphone, and the purpose of use (e,g. social media, web surfing, reading news, gaming, etc.). To identify ‘Addicted’ and ‘Non-Addicted’ participants the Smartphone Addiction Scale-Persian short Version SAS-SV-Pr was used. We assessed cervical flexor muscles endurance using the Neck Flexor Muscle Endurance Test (NFMET) [22], Neck extensor endurance using the Neck Extensor Muscle Endurance Test (NEMET), cervical position sense using a laser pointer custom-made device, and neck ROM using a manual goniometer.

### Neck Flexor Muscle Endurance Test (NFMET)

**Fig. 1:**
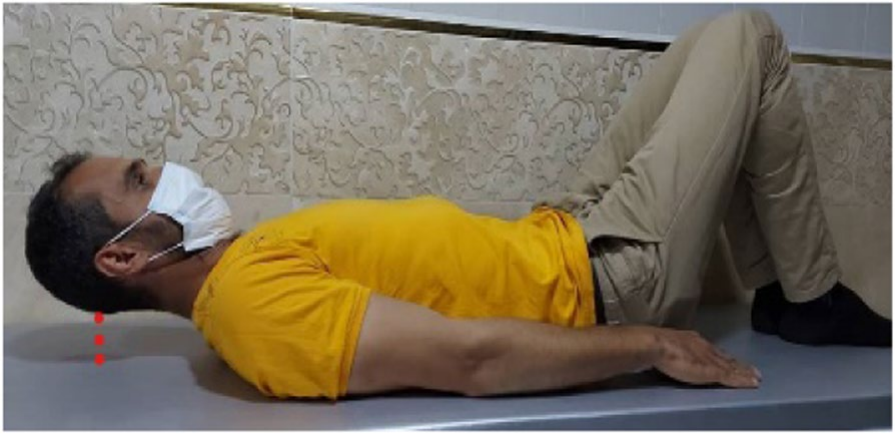
NFMET assessment

To perform this test, the subject is placed in a supine hook-lying position and performs a maximum isometric chin retraction, followed by a head and neck lift of approximately 2.5 cm above the table. While the subject maintains the chin tuck posture, the examiner puts their hand just below the occipital bone of the subject’s head (Fig. 1) and through verbal commands (‘Hold your head up’) encourages the subject to separate the head from the examiner’s hand and try as hard as possible to avoid touching the hand. When the subject is no longer able to maintain his/her head up and touches the hand, the test was completed (Fig. 1). The total length of time in seconds that the subject held the head in the ‘up position’ without touching the examiner’s hand is recorded by a stopwatch as the time for muscle endurance [22, 23].

### Neck Extensor Muscle Endurance Test (NEMET)

**Fig. 2:**
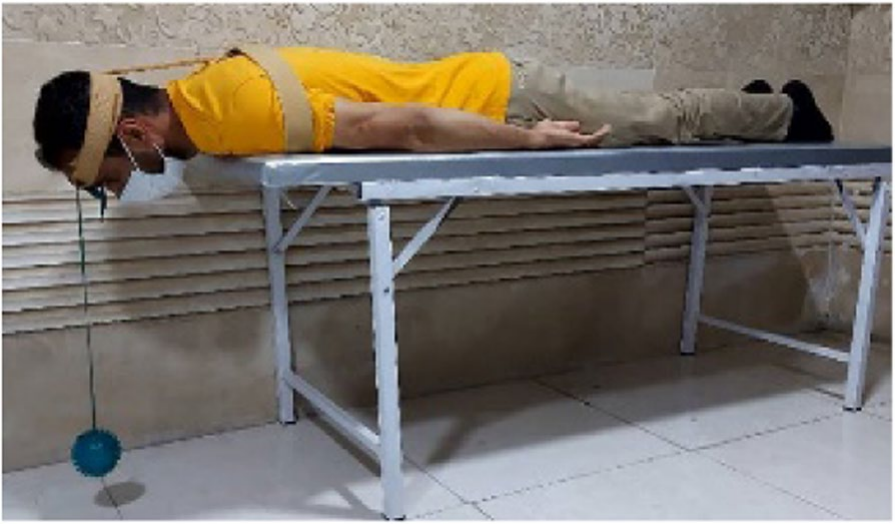
NEMET assessment

To perform this test, the procedure described by Ljungquist et al., was adopted [24]. The subject is lying in a prone position on the table while their arms are at their side and the head is over the end of the table and supported by the examiner (Fig. 2). To avoid the displacement of the thorax, it was supported by a strap. To monitor the head position, an inclinometer was attached to the posterior aspect of the head and fixed by a Velcro band. A 1-kg weight was suspended from the headband so that the weight was located just short of the floor. The subject's head was positioned in the neutral sagittal plane position and the cervical spine in a horizontal line with his/her chin. As the support of the examiner was removed the stopwatch recorded the time for the sustained position. The test was terminated when the weight returned to the floor or the neck horizontal position change more than 5° as shown by the inclinometer [23].

### Joint position sense (JPS) assessment

**Fig. 3:**
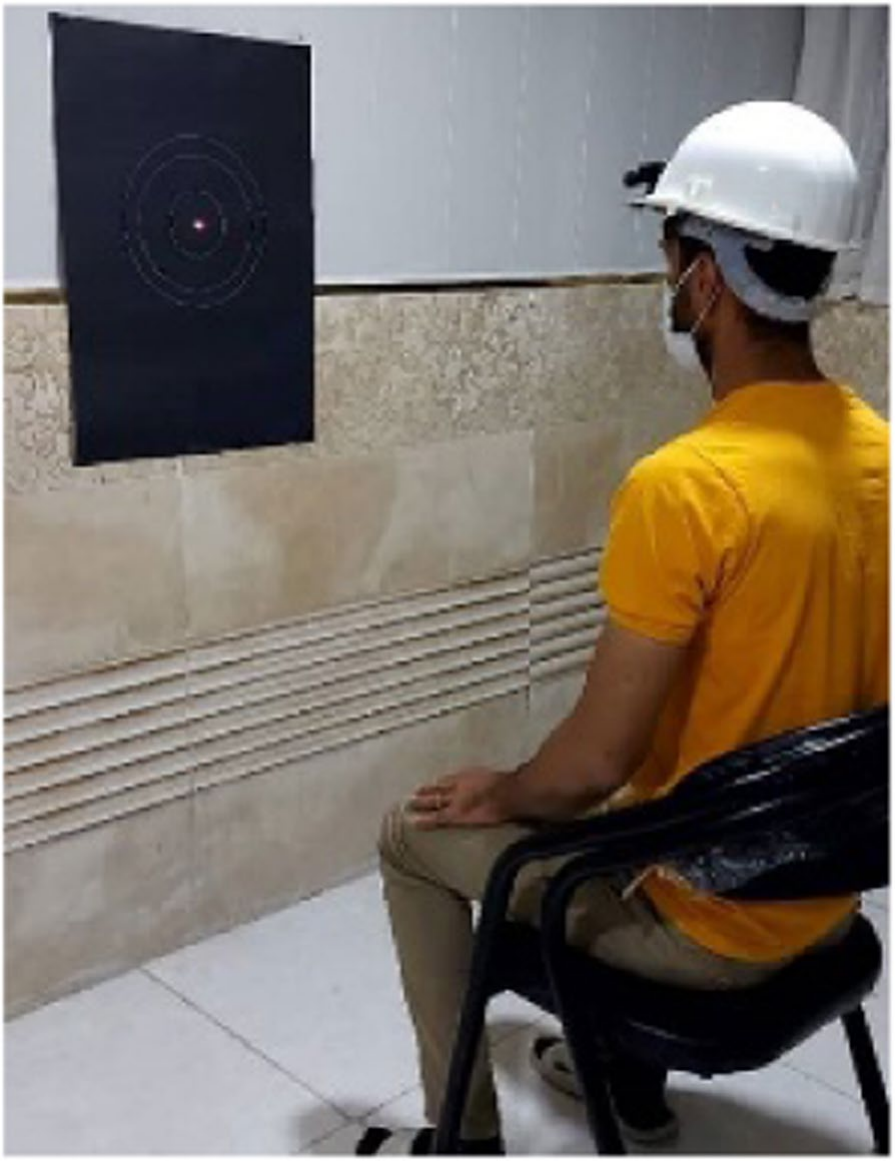
JPS assessment

In this test JPS was measured according to the experimental procedure presented by Revel et al. [25]. The ability to relocate the natural head posture (anatomic position) after performing either active cervical extension or left and right rotations, while blindfolded, was measured. The subject wears a light headpiece helmet strapped firmly to his/her head with a laser pointer fixed to the top of the helmet aimed at a target 90 cm in front of the subject. The subject was asked to sit in a comfortable position while their arms are hanging by their side, and the feet are on the floor. The eyes and vision were blocked by a sleeping mask and the subject is asked to assume a neutral position of the head and memorize it, and to be able to return to it after the completion of any movement. This point was considered as a reference point for each subject and after that the subject should actively perform maximum neck flexion and then return the head in the previous reference point (Fig. 3). The difference between the starting reference point and the returning point as determined by the laser beam on the target was recorded in cm as the error measure [26]. Three trials were performed for each direction (flexion, extension, lateral flexion), and the mean of the error difference was used in the statistical analysis.

### Cervical ROM (C-ROM)

Active cervical range-of-motion (ROM) was measured in the transverse (rotation), sagittal (flexion–extension), and frontal (lateral-bending) planes with a cervical ROM device. The test was performed by an occupational therapist with > 10 years of work experience. The C-ROM device was placed on the subject’s head while they were in an upright sitting position with both feet on the floor [26]. The difference between the start and end position in each direction from the natural posture was recorded as the ROM. Three repetitions were measured, and the average used for statistical analysis.

### Forward head posture (FHP)

**Fig. 4:**
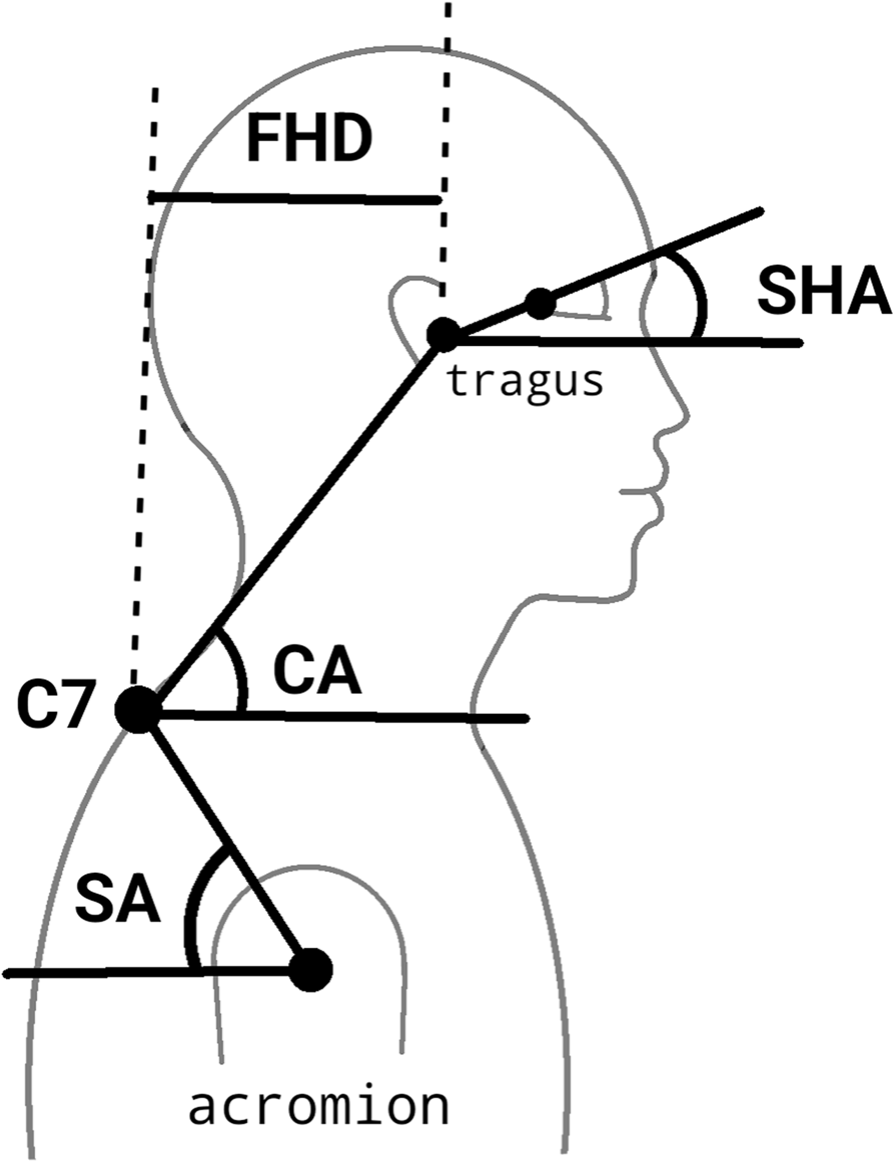
Analyzed parameters of the head and shoulder region. [29]. FHD = Forward head distance, SHA = Sagittal head angle, CA = Craniovertebral angle, SA = Shoulder angle, C7 = 7^th^ cervical spine vertebrae

A digital camera (Canon IXY 12, MP, Japan) was used to take a lateral view picture of the subject in standing and then calculate the relevant angles to assess the FHP [27]. The camera was fixed on a tripod at 1.5 m from the subject at the level of their shoulder [28]. The subject adopted a natural neck posture before the photography and for this purpose was asked to move their neck into flexion and extension and gradually reduced its range until the head and neck was placed in a natural self-selected comfortable position [27]. Three markers were attached: over the seventh cervical spinous process (C7); the tragus of the ear; and the acromion process. These were used to define the position of the head and neck in the sagittal plane. After taking the photograph, it was used for calculating the craniovertebral angle (CA), shoulder angle (SA), sagittal head angle (SHA) and forward head distance (FHD) as described below (Fig. 4) [29].

Craniovertebral angle (CVA) is the angle between the line joining the tragus of the ear to C7, and the horizontal line at C7, and has high reliability (*r* = 0.88) [30, 31]. A higher CVA indicated a lower FHP [32].

Shoulder angle (SA) is the angle between the line joining the C7 to the acromion process, and the horizontal line at the acromion process. A smaller SA represented a greater FHP and kyphotic posture [32].

Sagittal head angle (SHA) is the angle between the line joining the tragus of the ear to the canthus of the eye, and the horizontal line at the tragus. A lower value of this angle represented a lower FHP [32, 33].

Forward head distance (FHD) is the horizontal distance between the tragus, and the C7 vertebra. A higher distance indicated a higher FHP [27].

### Smartphone Addiction Scale-Short Version (SAS-SV)

The long form of the SAS is a 33-item questionnaire originally developed by Kwon et al. in 2013 [34]. Subsequently, a short version (SAS-SV) was presented with 10-items [35]. We used the validated Persian SAS-SV in this study to identify the level of the smartphone addiction risk and to distinguish high-risk participants. The psychometric properties of the Persian version were determined by Mokhtarinia et al. in 2020 [36] and demonstrated high reliability and validity. The cut-off values to detect the ‘Addicted’ person are: male = 31 and female = 33.

### Statistical analysis

All statistical analyses were performed using the SPSS statistical software version 16.0 (IBM SPSS Statistics for Windows). The descriptive results for quantitative variables were calculated as mean ± standard deviation (SD), and for categorical data as frequency and percentages. The normality of measurable data was evaluated using the Kolmogorov–Smirnov test. The sample did not follow a normal distribution; therefore, non-parametric statistical testing was performed. A Mann–Whitney test was used to determine whether there was a significant difference in measured variables between ‘Addicted’ and ‘Non-Addicted’ persons smartphone use. In controlling for the Type I error rate for the hypothesis testing, a Bonferroni correction was applied by dividing the significance level by the number of tested hypotheses (0.05:11 = 0.0045). The Spearman correlation coefficient was used to assess the associations and correlations between the variables.

## Results

### Participant characteristics

A total of 60 subjects were recruited into two *n* = 30 groups of 'Addicted’ (females = 14 and males = 16, aged 20–39 years) and ‘Non-Addicted’ (females = 14 and males = 16, aged 20–31 years). The subjects’ demographic characteristics are presented in Table 1.

**Table 1:** Demographic characteristics of subjects in two groups

	**Non-Addicted group (*n* = 30)**	**Addicted group (*n* = 30)**
	Mean ± SD (Range)	Mean ± SD (Range)
**Age (years)**	24.23 ± 3.51 (20–31)	24.93 ± 5.15 (20–39)
**Daily smartphone usage (hours)**	5.80 ± 2.56 (2–12)	6.56 ± 2.55 (2–15)
**Annual smartphone usage (years)**	6.43 ± 1.65 (2–8)	7.50 ± 1.57 (3–12)
**SAS score**	25.23 ± 5.50 (14–31)	43.90 ± 6.61 (34–54)
**Gender (F/M)**	14/16	14/16
**Marital status (single/marriage)**	26/4	26/4
**Education: BSc,**	17	14
**MSc, PhD**	30	16

Discomfort regions and pattern of use: The most common activities in which the participants reported discomfort when engage with their smartphones were social network usage (*n* = 57, 95%), followed by talking on the phone (*n* = 44, 73%), web surfing (*n* = 40, 66%), photography (*n* = 50, 30%), email usage (*n* = 24, 40%) and games (*n* = 12, 20%).

According to the reported discomfort regions during smartphone use, the most common were respectively the neck (*n* = 29, 48.3%), eyes (*n* = 28, 46.6), wrist and fingers (*n* = 32, 26.6%), and shoulders (*n* = 17, 28.3%).

#### Comparison between two groups

For the between-group comparison, the Mann–Whitney test showed a significant difference for extensor muscle endurance (*p* = 0.01), CA, and FHD (*p* < 0001) between the two groups. From these variables, CA and FHD remained statistically significant, however, Extensor Endurance was not significant after adjusting the alpha level for multiple comparisons to avoid a Type-I error using the Bonferonni method (0.05:11 = 0.0045). Table 2 shows descriptive statistics of the muscle endurance tests, ROM, JPS, FHD, SHA, CA, and SA measures, as well as the *p* values for between-group comparisons.

**Table 2:** Comparison results between two groups

Variable	Groups	Mean	SD	*P* value
SHA (Degree)	Non-Addicted	21.80	4.61	0.083
	Addicted	24.03	4.97	
CA (Degree)	Non-Addicted	54.70	4.89	< 0.001*^±^
	Addicted	47.66	3.47	
SA (Degree)	Non-Addicted	65.70	1.00	0.424
	Addicted	63.93	1.15	
FHD (cm)	Non-Addicted	9.40	1.67	< 0.001*^±^
	Addicted	11.86	1.71	
JPS (mm)	Non-Addicted	10.63	5.72	0.387
	Addicted	9.00	2.74	
Flexion ROM (degree)	Non-Addicted	50.90	9.46	0.911
	Addicted	51.43	8.23	
Extension (degree)	Non-Addicted	39.83	8.27	0.369
	Addicted	41.43	8.86	
Rotation (Degree)	Non-Addicted	41.86	8.10	0.296
	Addicted	41.70	7.57	
Lateral Flexion (Degree)	Non-Addicted	53.16	1.06	0.913
	Addicted	55.86	1.01	
Flexor Endurance (Seconds)	Non-Addicted	19.00	6.57	0.083
	Addicted	16.63	6.71	
Extensor Endurance (Seconds)	Non-Addicted	97.00	3.79	0.010*
	Addicted	74.86	2.23	

*FHD* Forward head distance, *SHA* Sagittal head angle, *CA* Craniovertebral angle, *SA* Shoulder angle, *JPS* joint position sense, *cm* centimeter, *mm* milimeter

^*^ *p* < 0.05 (significant before correcting α-level for multiple comparisons)

^±^*p* < 0.0045 (significant after correcting α-level for multiple comparisons)

#### Correlation analysis

*All data were pooled (n* = *60) and then a* Spearman analysis test showed a positive significant association between FHD and addiction level (*r* = 0.65; *p* < 0.001), and a negative association between CA and addiction level (*r* = -0.64; *p* < 0.001).

## Discussion

Postural changes and related discomfort, especially in the cervical region, in smartphone-addicted subjects are considered as a health problem that has dramatically increased, particularly during this global COVID-19 pandemic. There appears to be a lack of research into pathological smartphone-use that creates physiological disorders. A limited number of studies have evaluated the possible postural changes, muscle endurance, proprioception, and ROM changes in smartphone-addicted subjects.

In relation to FHP the results of the current study showed that there was a significant difference in head posture (i.e. CA and FHD) between the two groups of ‘Addicted’ and ‘Non-Addicted’ subjects. The CA and FHD are the two most common parameters for assessing FHP. In this study, the mean value of CA in 'Non-Addicted’ and ‘Addicted’ subjects were respectively 54.7 and 47.7 degrees. The normal value in healthy subjects is reported as ~ 52.7 which approximates our findings for the ‘Non-Addicted’ group [28]. The adoption of a flexed cervical spine posture during smartphone use induces the spine to being vulnerable to the FHP. In this posture, the subjects showed a larger FHD and a smaller CV angle than the neutral posture. In most smartphone tasks, the users shift the head forward (FHD increased) and downward to read or see the screen. As a result of this forward head shift, the lordosis of the lower cervical spine decreases and a compensatory thoracic kyphotic posture is created [37]. In the ‘Addicted’ group, because of prolonged and repetitive use of the smartphone, these changes are more probable, with the increased FDH and decreased CV angle expected. Similar to our results, Akodu et al., showed that CVA has a significant correlation with smartphone addiction [38].

In this study, however, no significant difference in mean SHA and SA between the two groups was found. The SA is correlated to ‘round shoulders’ postures. A higher SA represented a lower FHP and kyphotic posture. Further, SHA is related to the head tilt angle in which the lower the value of this angle, represented the lower FHP [32, 33]. In our results the mean value of SA in the ‘Addicted’ group was lower than the’Non-Addicted’, while the SHA was reversed, i.e. in the ‘Addicted’ it was higher than the ‘Non-Addicted’ group. Both parameters are indicative of higher FHP in the ‘Addicted’ group, however no significant difference was found.

For endurance our results showed that for the neck muscles this was lower in the ‘Addicted’ than ‘Non-Addicted’ subjects. However, the difference in extensor muscles was significant between the two groups before correcting the α-level for multiple comparisons. The mean annual smartphone usage was higher in the ‘Addicted’ than ‘Non-Addicted’ subjects and showed a negative significant correlation with the neck extensor endurance (*r* = -0.32, *p* = 0.01). It seems that significant reduction of neck extensor muscle endurance may develop due to the higher annual time of use of the smartphone. Lee et al., reported that there is a significant negative relationship between the duration of smartphone use and neck experienced discomfort [39]. In the commonly adopted postures during smartphone use, the neck is in flexion which places extra stress on the extensor muscles. It was reported that neck flexion from 15° to 60° increases the force on the neck from 12 to 27 kg [40]. The upper trapezius muscle has shown the highest and lowest muscle fatigue at 50° and 30° angles respectively [41]. Increased stress from the posture and duration of use can create a sense of discomfort in the extensor muscles which eventually results in neck pain. In parallel with symptoms reported by neck pain patients [42, 43], the addicted smartphone users may suffer from lower muscle endurance. Other studies have shown that smartphone use could be related to muscular fatigue and tenderness [44, 45]. Hence the reduced endurance results in our study can be explained because of the fatigability of the cervical extensor muscles due to prolonged use of smartphone. However, the neck extensor endurance did not pass the corrected significance threshold after allowing for the Bonferonni correction. This method of correction allows for multiple comparisons to be implemented, is very conservative, and has the potential to induce a Type-II error. Consequently the Neck muscle endurance should be considered as a measure of function or disability of the neck region [39]. We did not find a significant difference in endurance of the neck flexor muscles between the two groups. In agreement with our results, Can et al., showed no correlation between the endurance of the flexor muscles and smartphone addiction [46].

It is noted that participants of the smartphone-addicted group in this current study had not developed neck pain at the time of assessment. However, in the long-term the decreased endurance of the neck muscles should be monitored in a longitudinal study to clarify if musculoskeletal symptoms develop. For this purpose, education about the long-term ill effects of smartphone addiction and posture alteration during mobile and smartphone use, should be provided to the users [47].

For proprioception the adopted neck bending posture during smartphone use can cause damage to ligaments and soft tissue surrounding the cervical spine joints [48]. Neck proprioceptive receptors which are located mostly in the ligaments, muscles and related soft tissues are reflexive and viscoelastic. Long term use of the smartphone can affect the flexibility of these structures and incur pain and discomfort in the cervical region which in turn can impair proprioception [49]. Doland et al., has shown that adopting a slouched posture for 300 s resulted in significantly reduced lumbar spine reposition sense [49]. Similarly, as with Dolan’s study, Kim et al., showed that after 300 s smartphone use in the sitting posture, the reposition error of the upper and lower cervical spine were significantly increased [50]. Just one study has investigated the position sense in smartphone-addicted users in which a significant JPS difference was found between three levels of normal, moderate, and severe smartphone addiction. Lee et al., showed that the severe addiction group had the largest cervical vertebrae repositioning errors [48]. In comparison to the two first studies, we did not measure the position sense error through time variables. Hence our results are not comparable with those studies. However, we can conclude that long bending postures may be a risk factors for reduced JPS due to micro-injury or trauma in the soft tissue surrounding the joints.

In contrast to the results of Lee et al., JPS was not significantly different between our groups. The possible causes of obtained results are different outcome measures and a different category for addiction. In our study the score of 33 was considered as the cut-off point of smartphone addiction, while in the Lee’s study it is was notably higher at 43.

This study showed that the cervical ROM of the smartphone’Addicted’ group was not significantly different from the ‘Non-Addicted’ group. Cervical ROM has been evaluated in other samples such as visual display terminal (VDT) users [51], but to the best of our knowledge, only one study evaluated cervical ROM in smartphone users. Kee et al. [6] showed that smartphone addiction has a negative impact on the cervical ROM, which contrasts with our results.

### Limitations

This cross-sectional study design cannot establish cause-and-effect relationships between smartphone addiction and the measured parameters, and there may be a need for future longitudinal studies. In addition, possible gender differences need to be examined with a larger sample size in the same population (college students).

## Conclusions

Our results showed that in a smartphone group, the forward head posture is more common and extensor muscle endurance is likely to be decreased. Therefore, further studies are needed to investigate the long-term effects of smartphone usage on proprioception, muscle endurance and function in smartphone addicted subjects.

## Data Availability

Data is available on request due to privacy/ethical restrictions. The data that support the findings of this study are available on request from the corresponding authors, HRM. The data are not publicly available due to information that could potentially compromise the privacy of research participants.

## Abbreviations

FHP: Forward head posture
ROM: Range of motion
SAS-SV-Pr: Smartphone Addiction Scale-Persian short Version
NFMET: Neck Flexor Muscle Endurance Test
NEMET: Neck Extensor Muscle Endurance Test
JPS: Joint position sense
C-ROM: Cervical range-of-motion
C7: Seventh cervical
CVA: Craniovertebral angle
SA: Shoulder angle
SHA: Sagittal head angle
FHD: Forward head distance
F: Female
M: Male
VDT: Visual display terminal
